# Quantitative non-contrast T1 mapping of left ventricle in children and young adults

**DOI:** 10.1186/1532-429X-16-S1-P268

**Published:** 2014-01-16

**Authors:** Keyur Parekh, Cynthia K Rigsby, Roger A deFreitas, Bruce S Spottiswoode, Michael Markl

**Affiliations:** 1 Northwestern University, Chicago, Illinois, USA

## Background

Quantification of myocardial T1 values is a promising tool that may be used to quantify myocardial fibrosis. The purpose of our study is to establish a preliminary reference value of normal left ventricular (LV) myocardium in children and young adults using non-contrast T1 mapping.

## Methods

The HIPAA compliant prospective study was IRB approved. Sixteen patients (mean age 15.4 years; range 5-25 years) underwent cardiac magnetic resonance (CMR) (1.5-T) including non-contrast short axis T1 mapping (modified Look-Locker [MOLLI] sequence). Twelve patients with no intrinsic myocardial abnormality based on clinical history, standard biomarkers and echocardiographic criteria were compared with 3 patients having cardiac dysfunction and 1 patient with hypertrophic cardiomyopathy (HCM). Short axis images were manually contoured to outline the epicardium and endocardium using AHA 16-segment model yielding 192 normal myocardial segments, 48 segments in patients with cardiac dysfunction, and 16 segments in a patient with HCM. Patient groups were compared using one-way analysis of co-variance (ANOVA). Chi-squared test was performed to compare appropriateness of discrete data. Receiver operating characteristic (ROC) curve was used to obtain a cut-off value of T1 relaxation time. Statistical significance was defined as p < 0.05.

## Results

Myocardial T1 values in the normals (982.1 ± 30.0 ms) were significantly different (p < 0.001) when compared to patients with cardiac dysfunction (1021.0 ± 34.8 ms) and hypertrophic cardiomyopathy (1039.7 ± 20.8 ms). Non-contrast myocardial T1 cutoff of 1005 ms conceded a sensitivity of 80% and specificity of 83% when compared to patients with cardiomyopathy (p < 0.001).

## Conclusions

The normal non-contrast T1 values of LV myocardium in children and young adults obtained can be used as a baseline for comparison to patients with underlying myocardial abnormalities. Enrollment is underway to validate our results in a larger cohort.

## Funding

None.

**Figure 1:**
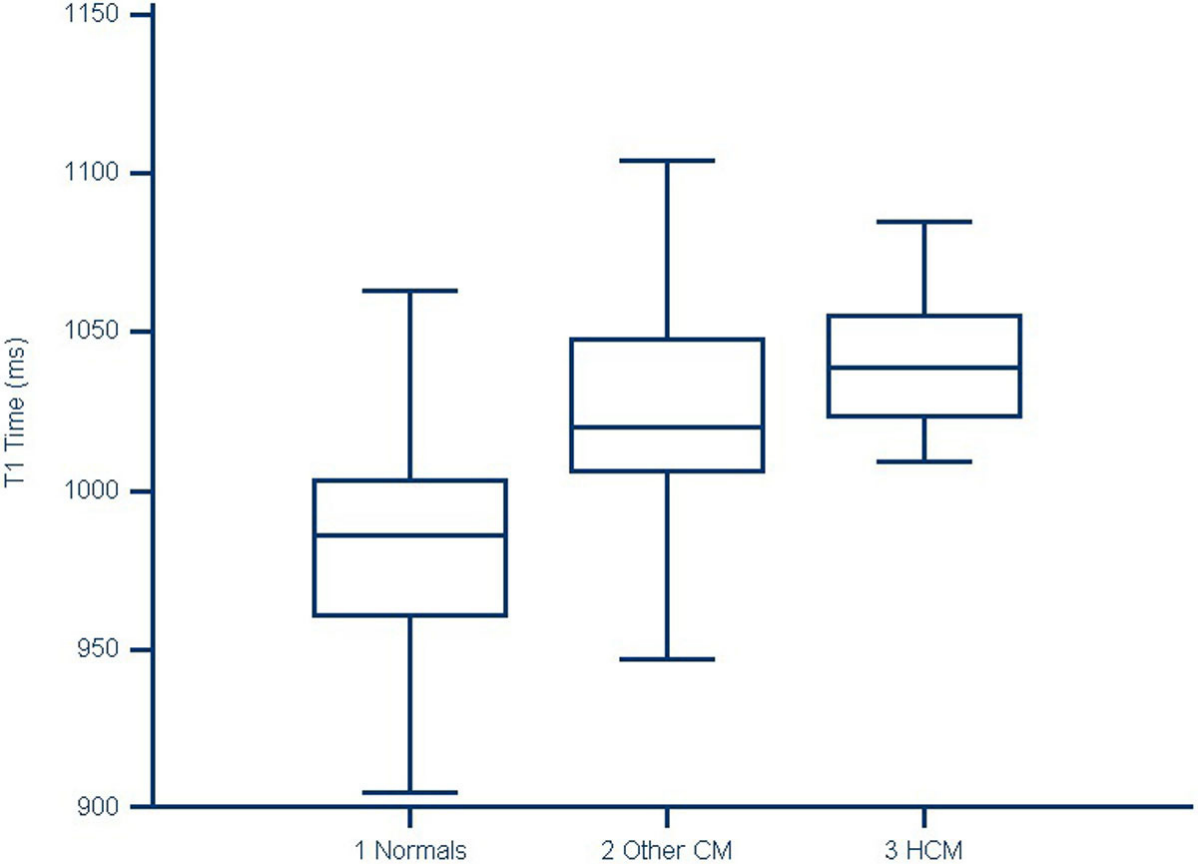
**Non-contrast myocardial T1 values in normal and cardiomyopathy (non-hypertrophic and hypertrophic) patients in children and young adults**. Mean values of left ventricular myocardium using non-contrast T1 MOLLI in normal population is 982.1 ± 30.0 ms. Non-hypertrophic cardiomyopathy (CM) and hypertrophic cardiomyopathy (HCM) has mean values of 1021.0 ± 34.8 ms and 1039.7 ± 20.8 ms. Values between these three groups were significantly different with p < 0.001.

**Figure 2:**
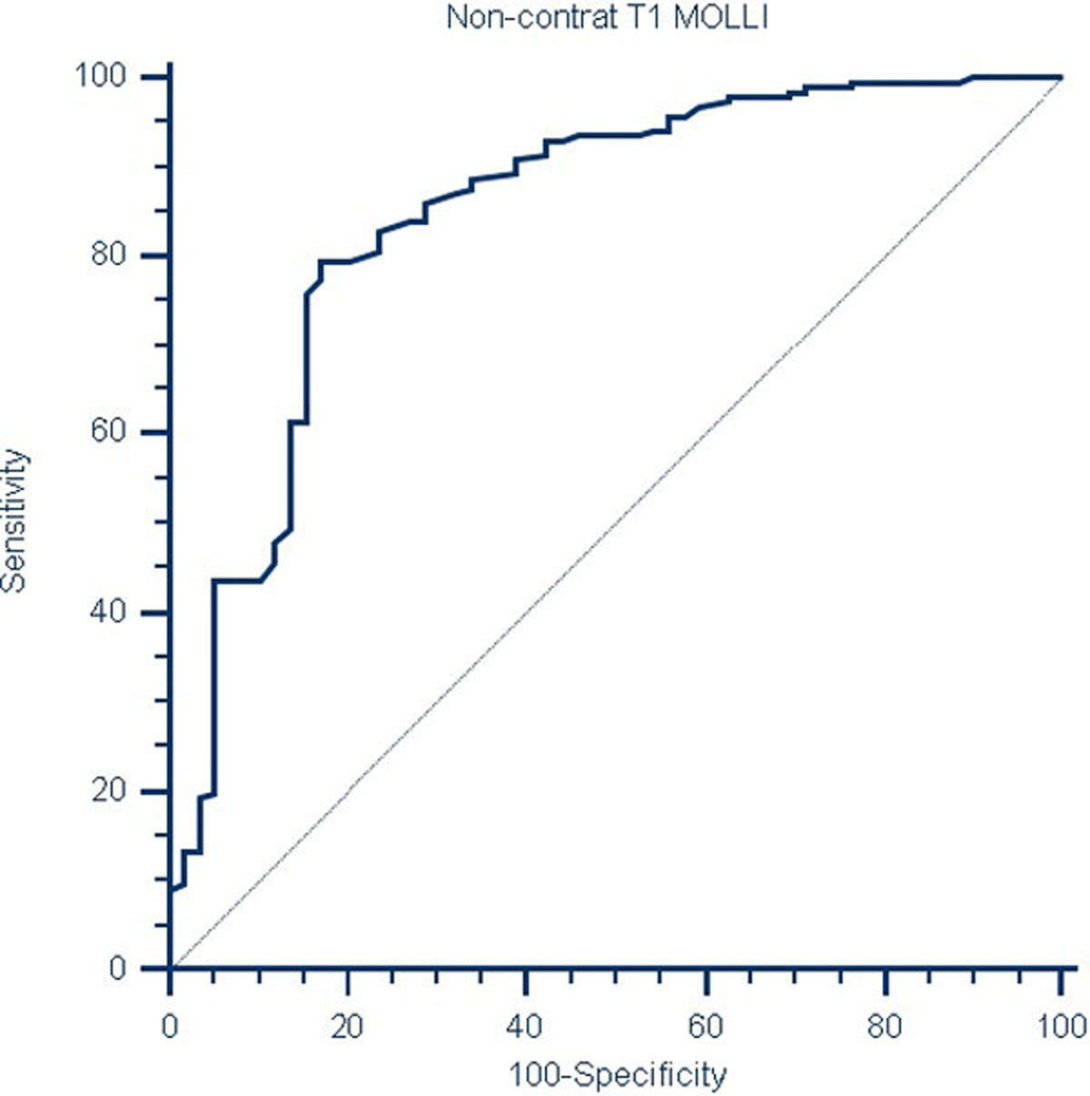
**Receiver-operating characteristic (ROC) curve to determine threshold value of T1 relaxation time of left ventricular myocardium**. Threshold of non-contrast T1 value to differentiate normal patients from patients with cardiomyopathy was calculated using ROC curve. Cut-off value of 1005 ms yielded sensitivity of 80% and specificity of 83% with area under curve (AUC) of 0.84 ± 0.03 (p < 0.001).

